# TRP Channels in Brain Tumors

**DOI:** 10.3389/fcell.2021.617801

**Published:** 2021-04-13

**Authors:** Giorgia Chinigò, Hélène Castel, Oana Chever, Dimitra Gkika

**Affiliations:** ^1^Laboratory of Cell Physiology, Department of Life Sciences, Univ. Lille, Inserm, U1003 – PHYCEL, University of Lille, Lille, France; ^2^Laboratory of Cellular and Molecular Angiogenesis, Department of Life Sciences and Systems Biology, University of Torino, Turin, Italy; ^3^UNIROUEN, Inserm U1239, DC2N, Normandie Université, Rouen, France; ^4^Institute for Research and Innovation in Biomedicine, Rouen, France; ^5^CNRS, Inserm, CHU Lille, Centre Oscar Lambret, UMR 9020-UMR 1277-Canther-Cancer Heterogeneity, Plasticity and Resistance to Therapies, University of Lille, Lille, France; ^6^Institut Universitaire de France, Paris, France

**Keywords:** ion channel, TRP channel, brain tumor, glioma, glioblastoma

## Abstract

Malignant glioma including glioblastoma (GBM) is the most common group of primary brain tumors. Despite standard optimized treatment consisting of extensive resection followed by radiotherapy/concomitant and adjuvant therapy, GBM remains one of the most aggressive human cancers. GBM is a typical example of intra-heterogeneity modeled by different micro-environmental situations, one of the main causes of resistance to conventional treatments. The resistance to treatment is associated with angiogenesis, hypoxic and necrotic tumor areas while heterogeneity would accumulate during glioma cell invasion, supporting recurrence. These complex mechanisms require a focus on potential new molecular actors to consider new treatment options for gliomas. Among emerging and underexplored targets, transient receptor potential (TRP) channels belonging to a superfamily of non-selective cation channels which play critical roles in the responses to a number of external stimuli from the external environment were found to be related to cancer development, including glioma. Here, we discuss the potential as biological markers of diagnosis and prognosis of TRPC6, TRPM8, TRPV4, or TRPV1/V2 being associated with glioma patient overall survival. TRPs-inducing common or distinct mechanisms associated with their Ca^2+^-channel permeability and/or kinase function were detailed as involving miRNA or secondary effector signaling cascades in turn controlling proliferation, cell cycle, apoptotic pathways, DNA repair, resistance to treatment as well as migration/invasion. These recent observations of the key role played by TRPs such as TRPC6 in GBM growth and invasiveness, TRPV2 in proliferation and glioma-stem cell differentiation and TRPM2 as channel carriers of cytotoxic chemotherapy within glioma cells, should offer new directions for innovation in treatment strategies of high-grade glioma as GBM to overcome high resistance and recurrence.

## Introduction

Malignant gliomas are the most prevalent group of primary brain tumors in adults, with an incidence of 8.9 cases per 100,000 persons/year in the US (Ostrom et al., 2015, 2017). Glioblastoma (GBM) remains one of the most aggressive human cancers. Glial tumors or glioma represent a wide spectrum of malignancies including grades II and III oligodendroglioma, grades II and III astrocytoma and glioblastoma from initial classification based on anatomocytopathological criteria related to the morphotypic characteristics and numerous cytonuclear atypologies, accompanied by anaplasia for high-grade glioma (GBM, grade IV) (Louis et al., 2007; Miller and Perry, 2007). The diagnosis of GBM was based on the presence of vascular micro-proliferations signs of intense vascularization, associated with zones of necrosis delimited by a hypoxic pseudopalissadic cellular zone, evidencing important intra-tumoral heterogeneity (Karsy et al., 2012; Alifieris and Trafalis, 2015). Now, brain tumors and glioma are classified according to the histomolecular classification recently published by the World Health Organization (WHO) (Louis et al., 2016), which represents a major clinical improvement for both diagnosis and treatments, as well as patient prognosis. The aim was to implement the histopathological classification with the following molecular signatures: first including the mutation in the isocitrate dehydrogenase1/2 (IDH1/2mut) and/or the co-deletion 1p/19q associated with oligodendroglioma or mutation of TP53; second the l′α-thalassemia mental retardation syndrome X-linked (ATX) or amplification of the epidermal growth factor receptor (EGFR) were associated with GBM IDHwt; finally the hypermethylation of the O6-methyl guanine-DNA methyl transferase (MGMT) promoter constitutes an important parameter of the GBM aggressiveness (Louis et al., 2016).

Despite these molecular attempts to stratify glioma patients with the objective to allow personalization of the treatments, the median survival of GBM patients currently ranges from 15 to 17 months, despite a safe maximal surgical resection, radiation/concomitant and adjuvant alkylating-based chemotherapy by temozolomide (TMZ) (Stupp et al., 2005, 2009; Wen and Brandes, 2009). However, more than 95% of GBM recur in the margin of the resection cavity, an area in which glioma tumor cells acting as a tumor reservoir are found (Giese et al., 2003). This invasiveness associated with X-ray and/or intrinsic or acquired chemoresistance of the glioma cells and the presence of an intrinsic or acquired blood-brain barrier (BBB), limit the effectiveness and/or the delivery of anti-neoplastic agents and justify the development of new strategies. In agreement, over the last decade, despite very important advances in the field of targeted therapy, none of them, e.g., drug/antibody or combination of small molecule inhibitors, has been shown to be more effective than TMZ or capable of increasing the efficacy of standard therapy in patients with primary or recurrent GBM (Stepanenko and Chekhonin, 2018), and no curative treatment is currently identified in GBM. This failure may be explained at least in part by the intratumoral heterogeneity which is a conserved consequence of the GBM micro-environment (Prabhu et al., 2017), referring to the physico-chemical characteristics and matrices interacting with the tumor. Indeed, the GBM heterogeneity is tightly related to angiogenic and hypoxic features as well as invasive processes, thus future strategies should consider targeting mechanisms associated with resistance and invasion. In particular, Watkins et al. (2014) showed that GBM cells can thus control the regulation of vascular tone, *via* the release of K^+^ through K^+^ channels activated in response to Ca^2+^, leading to an adaptation of cell volume to facilitate their invasion.

Transient receptor potential (TRP) channels are a superfamily of cationic tetrameric channels, mostly permeable to Ca^2+^, involved in various physiological functions, and for the most part sustain calcium homeostasis and calcium signaling. Calcium-dependent mechanisms determine several aspects of brain tumor cell homeostasis including survival, proliferation, invasion or treatment resistance, making TRP channels putative potent modulators of tumorigenesis and glioma progression. Approximately 30 TRPs have been identified and are classified into TRPA (ankyrin family), TRPC (canonical family), TRPM (melastatin family), TRPN (NomPC family), TRPML (mucolipin family), TRPP (polycystin family), and TRPV (vanilloid family) (Li, 2017). These cationic channels have been shown to be gated by many physical or chemical stimuli (temperature, membrane potential, pH, hormones, vitamins …). TRP channels are expressed in various excitable and non-excitable cell types and are present in many organs, including brain, heart, liver, lung, kidney, spleen, muscle, skin, pancreas (Venkatachalam and Montell, 2007). Since a decade, TRP channels have attracted much interest in the cancer field and tumorigenesis. Activities of TRP channels have been linked to cell growth, survival or migration, being involved in a plethora of cancers, especially for TRPC, TRPM, and TRPV (Prevarskaya et al., 2007; Gkika and Prevarskaya, 2009; Fiorio Pla and Gkika, 2013). Recent research unravels the role of some TRP channels in glioma growth and progression or glioma stem-like cell fate determination. In this review, we will mainly focus on a new class of molecular players, TRP channels emerging in gliomas and for which we will develop three aspects: (i) the expression profile and use as clinical markers; (ii) the molecular mechanisms through which they act; and (iii) their potential use in therapeutics.

## Transient Receptor Potential (TRP) Expression Profile and Putative Biomarkers

Changes in expression of TRP channels have been related to cancer development and progression, thus making them valuable diagnostic and/or prognostic markers in several tumor types, including glioma. Furthermore, a strong correlation between clinical-pathological findings and mRNA and/or protein expression of different TRPs has been recently provided. For instance, mRNA encoding TRPC1, TRPC6, TRPM7, TRPM8, TRPV4, and TRPML2 appeared up-regulated in GBM tumor specimens in comparison with normal tissues and their expression was found to increase with glioma tumor grade with the highest mRNA level found in GBM patient samples (Ding et al., 2010; Alptekin et al., 2015). These findings are consistent with a pro-tumorigenic role of these channels in glioma progression and aggressiveness, as described in more detail in the next paragraph. According to a qPCR screening of 33 GBM patient tumors, additional mRNA-encoding TRP channels including TRPM2, TRPM3, TRPV1, TRPV2 showed significantly higher expression levels in GBM compared with control normal brain tissues (Alptekin et al., 2015). However, other studies reported opposite results, more consistent with an anti-tumorigenic function of these channels, as confirmed by several experimental data (Amantini et al., 2007; Nabissi et al., 2010; Ying et al., 2013; Morelli et al., 2019). These discrepancies could be due to the relatively low number of patients considered in the first study (Alptekin et al., 2015), which may not be very representative enough while further investigations should now be reconsidered in light of the new molecular GBM patient stratification and the methylome.

Among more than 20 TRP channels investigated, TRPM8 showed the highest mRNA upregulation in GBM as compared with normal brain tissue (Alptekin et al., 2015; Zeng et al., 2019), suggesting a pivotal function of TRPM8 in gliomagenesis. Moreover, TRPM8 expression in human GBM specimens and established GBM cell lines was found to be up-regulated at both mRNA and protein level to a variable extent (Klumpp et al., 2017) and to be significantly correlated with worse patient overall survival (Zeng et al., 2019). Interestingly, it was previously reported that TRPM8 is a primary androgen-responsive gene since its promoter is located downstream an androgen response elements (AREs) to which androgen receptor (AR) may bind once activated by androgens thus promoting TRPM8 expression (Bidaux et al., 2005; Asuthkar et al., 2015). Therefore, TRPM8 overexpression in high-grade glioma might be associated with the documented upregulation of AR in GBM (Yu et al., 2015). Moreover, it has been recently found that AR may also directly regulate TRPM8 channel activity via protein-protein interaction and/or TRPM8 phosphorylation, further accentuating the close TRPM8-androgens relationship (Grolez et al., 2019a; Gkika et al., 2020). Similarly to TRPM8, TRPV4 has been shown to positively correlate with glioma progression, since high levels of TRPV4 gene and protein expression were associated with a poorer patient prognosis (Ou-yang et al., 2018). Thus, TRPM8 and TRPV4 may be currently considered promising biomarkers accompanying aggressiveness of glioma and signature of GBM while constituting potential therapeutic targets for future treatment options. TRPML2 expression was also detected in normal astrocytes and neural stem/progenitor cells and to be up-regulated at both mRNA and protein level in glioma to a variable extent, increasing with the pathological grade (Morelli et al., 2016). Such observation was linked to the up-regulation of the transcriptional activator of the TRPML-2 gene Paired box 5 (PAX5) (Valadez and Cuajungco, 2015) found in human astrocytoma and correlated with malignancy and pathological grade of glioma (Stuart et al., 1995).

Moreover, the loss of TRPM3, TRPV1, TRPV2, and TRPML1 expression has been proposed as a negative prognostic marker for GBM patients, because of their significant and progressive down-regulation as the tumor grade increases. For instance, a study focusing on the role of miR-204 in high-grade glioma cell lines has revealed a significant down-regulation of TRPM3, due to the hypermethylation of its promoter (Ying et al., 2013). Interestingly, miR-204 is an intronic miRNA located between exons 7 and 8 of the TRPM3 gene and its loss in glioma, due to the high methylation of its host gene TRPM3, is associated with an enhancement in cell migration and cellular stemness (Ying et al., 2013) questioning the direct role of TRPM3 and the indirect regulatory functions of miR-204 *via* its target genes. Consistently, restoration of miR-204 in LN382T and SNB19 cells orthotopically xenografted in the brains of nude mice suppressed tumorigenesis and invasiveness and increased animal survival (Ying et al., 2013). Taken together, these findings might suggest a potential tumor-suppressive function of TRPM3 in glioma, but further studies are required to clarify its involvement in cancer development and/or progression and to establish whether TRPM3 and miR-204 might cooperate with each other in the pathogenesis of gliomas. Concerning TRPV1 and TRPV2, the preventing role in gliomagenesis and tumor progression has been more clearly established and characterized (see next paragraph). First, TRPV1 and TRPV2 genes and protein expression appeared inversely correlated with glioma grade, showing an almost undetectable level in GBM (Amantini et al., 2007; Nabissi et al., 2010; Morelli et al., 2016). In particular, a study performed by Nabissi and coworkers has shown that GBM and glioma stem-like cells (GSC) selectively express the TRPV1 5′-untranslated region (5′UTR) variant three (TRPV1_*v*__3_), one of the four variants resulting from alternative first exon splicing (Nabissi et al., 2016). The 5′UTR can generate different transcripts encoding the same protein but characterized by different stability and translation efficacy (Audic and Hartley, 2004; Gebauer and Hentze, 2004; Hinnebusch et al., 2016) and TRPV1_*v*__3_ is the most stable TRPV1 5′UTR transcript. In GBM, the mRNA expression of the unique TRPV1_*v*__3_ variant correlates with the patient’s survival, suggesting that its loss or low mRNA expression may represent a potential marker of poor prognosis in GBM patients (Nabissi et al., 2016). Similarly, the clinical relevance of the overexpression of TRPV2 in GBM was confirmed through the analysis of the TRPV2-interactome based signature using a systematic proteomics and computational analysis approach (Doñate-Macián et al., 2018), predicting GBM patient overall survival. Indeed, high TRPV2 interactome protein expression was correlated with tumor progression, recurrence, TMZ-resistance and a poor prognosis (Doñate-Macián et al., 2018). Finally, also TRPML1 might have a potential role as a negative prognostic marker for GBM patients (Morelli et al., 2019) since TRPML1 mRNA down-regulation or loss strongly correlates with reduced overall survival in GBM patients. However, additional studies are needed in order to further investigate the relationship between TRPML1 expression and lower glioma grades (Morelli et al., 2019). However, TRPML1 expression at mRNA and protein levels displayed variability within patient samples and its subcellular localization may also be distinct since TRPML1 is mainly expressed in the late endosome/lysosome of normal cells while found in endolysosomes and as dot spots in the nuclear cell compartment in glioma cells (Morelli et al., 2019). The mechanisms underlying this nuclear localization in tumor cells and the effects of this specific localization are not completely characterized, although it has been shown that TRPML1 is able to bind DNA somehow and thus, it might affect the transcription of some genes involved in tumor progression (Morelli et al., 2019).

Together, these studies highlight that some TRPM, TRPV, and/or TRPML channels overexpressed in glioma should be considered as predictive and specific biomarkers of high-grade glioma and GBM, and through changes in their permeability to cations they may play a role in GBM aggressiveness.

## Molecular Mechanisms of TRP Channels Action

**FIGURE 1:**
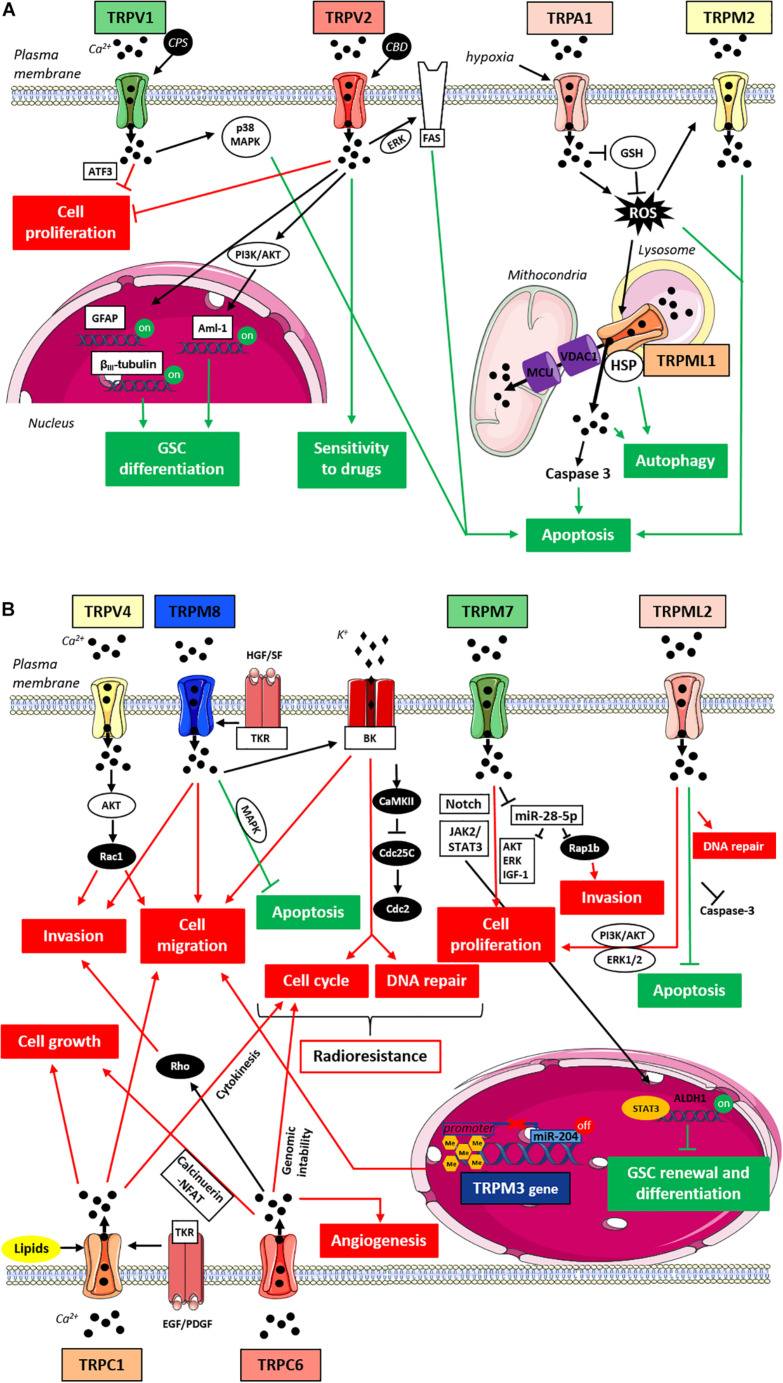
TRPs-mediated signaling pathways in gliomas. **(A)** Anti-tumorigenic TRPs-mediated signaling pathways in gliomas. Cartoon depicting TRP channels signaling pathways suppressing gliomas progression through the inhibition of pro-tumorigenic pathways (in red) and/or the activation of anti-tumorigenic signaling pathways (in green). TRPM2 and TRPML1 upon ROS activation promote cell apoptosis through an increase in oxidative stress and intracellular Ca^2+^ concentration; due to its localization on the lysosomal membrane TRPML1 may also mediates the autophagic cell death pathway through the interaction with HSP; TRPA1 under hypoxia promotes apoptosis inducing ROS formation and inhibiting antioxidants such as GSH; TRPV1 upon stimulation with CPS triggers the apoptotic pathway through p38 MAPK and reduces glioma expansion via ER pathway in a ATF3-dependent manner; TRPV2 reduces cell proliferation and enhances the Fas-induced apoptosis in an ERK-dependent manner; TRPV2 also acts on cell differentiation since its overexpression is associated with GFAP and βIII-tubulin increased expression and its activation by CBD promotes Aml-1 up-regulation via PI3K/AKT pathway; finally, TRPV2 activation by CBD can improve cell sensitivity to chemotherapeutic agents favoring drug uptake. **(B)** Pro-tumorigenic TRPs-mediated signaling pathways in gliomas. Cartoon depicting TRP channels signaling pathways promoting gliomas progression through the activation of pro-tumorigenic pathways (in red) and/or the inhibition of anti-tumorigenic signaling pathways (in green). TRPC1 affects cell growth and cell proliferation mainly promoting cytokinesis in response to lipid activation, whereas upon stimulation with growth factors it induces chemotactic migration; TRPC6 affects cell proliferation, tumor growth and angiogenesis likely through the Ca^2+^-induced activation of the calcineurin-NFAT pathway, whereas TRPC6 effects on cell migration might rather involve Rho activation and subsequent actin cytoskeleton rearrangements; TRPV4 promotes cell migration and tumor invasiveness through the AKT-mediated Rac1 activation; TRPM8 supports glioma progression by inhibiting apoptosis through MAPK pathway and impairing the cell cycle through the activation of BK channels and the subsequent CaMKII-mediated inhibition of phosphatases like Cdc25C and Cdc2; TRPM8 effects on cell migration/invasion are also associated with BK channels activation and the function of TRPM8 in aggressiveness and resistance to treatment may also be potentiated by TKR-mediated HGF/SF stimulation; TRPM7 increases glioma cell proliferation and invasion through the down-regulation of miR-28-5p and the subsequent up-regulation of oncogenic signaling pathways involving AKT, ERK, IGF-1 and Rap1b; TRPM7 effects on glioma proliferation may also be mediated by Notch and/or JAK2/STAT3 signaling pathways and through the activation of STAT3; TRPM7 might also be involved in GSC renewal and differentiation thanks to the up-regulation of the well-known GSC marker ALDH1; the hypermethylation of TRPM3 promoter through the down-regulation of miR-204 enhances cell migration; TRPML2 enhances cell proliferation and slows down apoptosis improving DNA repair and inhibiting Caspase 3 likely through PI3K/AKT and ERK1/2 pathways. Abbreviations: ROS, reactive oxygen species; HSP, heat shock proteins; GSH, glutathione; CPS, capsaicin; ATF3, transcription factor-3; CBD, cannabidiol; GFAP, glial fibrillary acidic protein; Aml-1, acute myeloid leukemia transcription factors; PI3K, phosphoinositide 3-kinases; AKT, protein-kinase B; HGF/SF, hepatocyte growth factor/scatter factor; EGF, epidermal growth factor; PDGF, platelet-derived growth factor; TKR, tyrosine kinase receptor; NFAT, nuclear factor of activated T-cells; MAPK, mitogen-activated protein kinase; BK, big potassium channels; CaMKII, Ca^2+^/calmodulin-dependent protein kinase II; Cdc25C and Cdc2, cell division cycle proteins; JAK2, janus kinase 2; STAT3, signal transducer and activator of transcription 3; ERK, extracellular signal-regulated kinases; IGF-1, insulin-like growth factor-1; GSC, glioma stem cells; ALDH1, aldehyde dehydrogenase 1.

Transient receptor potential channels have revealed a direct involvement in determining many hallmarks of glioma and GBM (Table 1), including some typical histological cellular abnormalities (Bomben and Sontheimer, 2008), its relentless growth, and its intrinsic severe aggressiveness due to its high capability to diffuse into the non-neoplastic brain parenchyma, which contributes to treatments resistance and bad prognosis (Demuth and Berens, 2004; Schwartzbaum et al., 2006; Liu et al., 2018). TRP channels may exert both anti-tumorigenic and pro-tumorigenic functions in gliomas and the main TRPs-mediated signaling pathways associated with gliomas progression are schematically summarized in Figure 1. Most of the TRP channels involved in gliomagenesis and tumor progression were found to affect more than one cellular process related to carcinogenesis. In this chapter, we will therefore discuss the molecular mechanisms by which each TRP affects cancer cell behavior, subgrouping them into subfamilies.

**TABLE 1:** TRP channels expression and functionality in gliomas/glioblastomas.

	Expression					
Channel	Healthy	Tumor progression	Biological effect	Mechanism	Potential therapeutic tools	References
TRPC1	Yes	↑	Cell growth (+)	↑ Cytokinesis	TRPC1/SPK/PI3K inhibitors	Bomben and Sontheimer, 2008; Bomben and Sontheimer, 2010
			Cell migration (+)	↑Chemotaxis		Bomben et al., 2011; Lepannetier et al., 2016
TRPC6	Low	↑	Cell growth (+)	→ NFAT	TRPC6/NFAT inhibitors	Chigurupati et al., 2010; Ding et al., 2010
			Cell migration (+)	→ RhoA		Chigurupati et al., 2010
			Angiogenesis (+)	→ NFAT		
			Radioresistance (+)	↑ G_2_/M (Cdc25C)		Ding et al., 2010
TRPM2	Yes	↑	Cell death (+)	↑ ROS-induced Ca^2+^ influx	TRPM2 gene insertion (+ Se and DXT)	Ishii et al., 2007; Ertilav et al., 2019
TRPM3	Yes	↑/↓	n.d.	n.d.	n.d.	Ying et al., 2013; Alptekin et al., 2015
TRPM7	Yes	↑	Cell growth (+)	↑ STAT3/Notch	TRPM7/STAT3/Notch/ALDH1 inhibitors miR-28-5p	Liu et al., 2014; Wan et al., 2019
				⊣miR-28-5p ⊣ Rap1b		
			Cell invasion (+)	⊣miR-28-5p ⊣ Rap1b		Wan et al., 2019
			Stem cell renewal and differentiation (+)	STAT3-ALDH1 ↑		Liu et al., 2014
TRPM8	Yes	↑	Cell migration (+)	↑ BK channels RTK signaling		Wondergem et al., 2008; Wondergem and Bartley, 2009; Klumpp et al., 2017
		(2 different isoforms)	Cell growth (+)	→ BK channels → CaMKII ⊣cdc25C ⊣ cdc2 MAPK signaling		Klumpp et al., 2017; Zeng et al., 2019
			Cell death (–)	MAPK signaling		Klumpp et al., 2017; Zeng et al., 2019
			Radioresistance (+)	Supporting DNA repair and cell cycle upon genotoxic stress		Klumpp et al., 2017
TRPV1	Yes	↓	Apoptosis (+)	→ p38 MAPK		Amantini et al., 2007
				↑ ER stress (ATF3)		Stock et al., 2012
TRPV2	Yes	↓	Cell proliferation (–)	ERK signaling		Nabissi et al., 2010; Morelli et al., 2012
			Apoptosis (+)	Fas signaling		Nabissi et al., 2010
			Cell differentiation (+)	↑ GFAP and β_*III*_-tubulin expression ↑ Aml-1 a (PI3K/AKT pathway)	CBD	Morelli et al., 2012; Nabissi et al., 2015
			Drug sensitivity (+)	↑ drug uptake ↑ drug-mediated apoptotic pathway	CBD + TMZ/BCNU/DOXO	Nabissi et al., 2013, 2015
TRPV4	Yes	↑	Cell migration (+)	AKT/Rac1 signaling	TRPV4 inhibitors (HC- 067047)	Ou-yang et al., 2018
TRPA1	n.d.	n.d.	Cell apoptosis (+)	↑ mitochondrial stress	TRPA1 activators	Deveci et al., 2019
TRPML1	Yes	↓	Cell apoptosis (+) Autophagy (+)			Morelli et al., 2019
TRPML2	Yes	↑	Cell proliferation (+)	PI3K/AKT – ERK 1/2 signaling		Morelli et al., 2016
			Cell apoptosis (–)			

*Abbreviations: +, increase; −, decrease; ↑, increment; ↓, reduction; →, activation; ⊣, inhibition. SPK, sphingosine kinase; PI3K, phosphoinositide-3 kinase; NFAT, nuclear factor of activated T-cells; Se, selenium; DXT, docetaxel; STAT3, signal transducer and activator of transcription 3; miR-28-5p, miRNA 28-5p; ALDH1, aldehyde dehydrogenase1; BK, large-conductance Ca^2+^-activated K^+^ membrane ion channels; RTK, Met receptor tyrosine kinase; CaMKII, Ca^2+^/calmodulin-dependent protein kinase II; Cdc25C, M-phase inducer phosphatase 3; Cdc2, subunit of the M phase-promoting factor; MAPK, mitogen-activated protein kinase; ATF3, transcription factor-3; CBD, cannabidiol; ERK, extracellular signal-regulated kinase 1/2; GFAP, glial fibrillary acidic protein; Aml-1 a, acute myeloid leukemia variant a; AKT, protein kinase B; TMZ, temozolomide; BCNU, carmustine; DOXO, doxorubicin. n.d., not determined.*

### Canonical TRPs

It has been suggested that TRPC channels-relayed mechanisms may contribute to some of the most common histopathological hallmarks of GBM such as nuclear atypia and enlarged cell shape (Bomben and Sontheimer, 2008). Glioma cell lines and surgical patient-derived tumors have revealed the expression of four TRP channels belonging to the TRP canonical subfamily that are TRPC1, TRPC3, TRPC5, and TRPC6. Further investigations on their role in glioma cells have suggested an involvement of these channels in a Ca^2+^ influx pathway impacting cellular growth. More specifically, it has been shown that TRPC channels contribute to the resting conductance of glioma cells while their acute pharmacological inhibition with SKF96365 increased membrane resistance of glioma cells and caused a transient hyperpolarization followed by a sustain depolarization of the cells’ membrane (Bomben and Sontheimer, 2008). Additionally, chronic application of the TRPC inhibitor SKF96365 (from 0 to 5 days) would lead to an almost complete growth arrest at the G_2_/M phase of GBM D54MG cell cycle, as revealed by FACS analysis (Bomben and Sontheimer, 2008). In most cases the blockage of the cell cycle during the G_2_/M transition leads to cell death (Stark and Taylor, 2006), or in the particular case of D54MG cells, TRPC inhibition was accompanied by a continued growth exhibiting multinuclear and enlarged cells due to incomplete cytokinesis. This phenotype might render impossible a dynamic adaptation of the cell volume to invade the brain parenchyma, through the narrow extracellular brain spaces compatible with less recurrence.

Glioma cells display a depolarized resting membrane potential around –30 mV. Some TRPC channels are opened at rest and contribute to this membrane potentials (Bomben and Sontheimer, 2008). Activation of TRPC channels can also lead to membrane potentials fluctuations by various ways. First of all, TRPC channels are non-selective cation channels and they directly depolarize cells following activation. In addition, TRP channels are functionally coupled with other ion channels, so their activation can indirectly lead to depolarizations or hyperpolarizations, depending on the channels involved (Gees et al., 2010). In particular, major conductances of glioma cells are mediated by Ca^2+^-activated K^+^ channels (Ransom et al., 2001) and Cl^–^ channels such as ClC3 or ClC2 (Mcferrin and Sontheimer, 2006), which both are modulated by TRPC channels. Glioma cells do express many types of channels and transporters (Cuddapah and Sontheimer, 2011; Molenaar, 2011), which are sensitive to membrane potential fluctuations. Furthermore, glioma cells have been shown to display electrical activities similar to Na^+^ spikes, which are sustained by TTX-sensitive voltage-gated Na^+^ channels (Bordey and Sontheimer, 1998). Thus, TRPC channels may impact many voltage-dependent cellular processes and modulate electrical behavior of glioma cells.

Among the TRPCs, TRPC1, and TRPC6 are those for which the mechanism of action has been best characterized in human malignant glioma. For instance, TRPC1 was found to act on several hallmarks of cancer, including growth, cell cycle and migration. Interestingly, all the TRPC1 effects on glioma cell behavior strictly resulted from lipid regulation, e.g., in some cases the channel activity is directly affected by lipids [phosphatidylinositol-(4,5)-bisphosphate PIP_2_, phosphoinositides, diacylglycerol, cholesterol, etc.] localized on the plasma membrane, in other cases, it can be by signaling pathways which lead to the production of specific lipids such as sphingolipids. It has been shown that the loss of TRPC1-mediated Ca^2+^ influx upon pharmacological inhibition or constitutive/inducible shRNA silencing, is associated with reduced cell proliferation and incomplete cell division, thus resulting in multinucleated cells similar to those found in patient biopsies (Bomben and Sontheimer, 2010). The important role of TRPC1 in glioma cell division has been also confirmed *in vivo* through a shRNA knockdown approach on a flank GBM cell tumor model: TRPC1 downregulation led to a significant decrease in tumor size, most likely impairing calcium signaling during cytokinesis (late M-phase) (Bomben and Sontheimer, 2010). TRPC1 has also revealed a role in controlling glioma cell migration. In particular, it has been shown that TRPC1 is essential for chemotactic migration in human malignant gliomas in response to chemoattractant growth factors like epidermal growth factor (EGF) and platelet-derived growth factor (PDGF) which affect TRPC1 activity through different signaling pathways (Bomben et al., 2011; Lepannetier et al., 2016). Stimulation with EGF was associated with a re-localization of TRPC1 channel at the leading edge of migrating D54MG glioma cells within lipid rafts, specialized membrane microdomains enriched in cholesterol and sphingolipids (Mollinedo and Gajate, 2015). In agreement, it has been shown that both TRPC1 channel activity and lipid raft integrity were required for gliomas chemotaxis (Bomben et al., 2011). Moreover, the disruption of lipid rafts by depletion of cholesterol not only affected chemotaxis but also impaired TRPC currents in whole-cell recordings and decreased store-operated Ca^2+^ entry (SOCE), confirming a direct interplay between lipid rafts and TRPC1 channels and localized Ca^2+^ rise in regulating the chemotactic movement of glioma cells (Bomben et al., 2011). It must be noted that TRPC pharmacological inhibition through non-selective inhibitors caused an almost complete loss of chemotactic migration but TRPC1 knockdown through shRNA compromised directional migration but did not eliminate it and did not affect non-directional motility. This suggests TRPC1 specific implication in chemotactic migration and the potential implication of other TRPC channels in migration processes (Bomben et al., 2011). As shown by a more recent study by Lepannetier et al. (2016), lipids are not only important for the regulation of TRPC1 at the membrane level, but also through their signaling. The authors shed light on another store-independent mechanism by which TRPC1 may be activated and thus affect cell migration in GBM. In particular, they have shown that PDGF may induce the translocation of TRPC1 from the cytosolic compartment to the front of migrating cells through a mechanism requiring the phosphoinositide-3 kinase (PI3K) and at the same time induces the production of the lipid second messenger sphingosine-1-P (S1P) which in turn, activates TRPC1-mediated Ca^2+^ entry. Indeed, the PDGF-induced Ca^2+^ influx through TRPC1 can be partially inhibited by pretreatment of the cells with a specific inhibitor of the sphingosine kinase (SPK) producing S1P (Lepannetier et al., 2016). However, whether S1P directly or indirectly triggers TRPC1-mediated and store-independent entry of Ca^2+^ channel remains to be clarified (Lepannetier et al., 2016). In any case, it has been well established that both TRPC1 targeting to the leading edge of lamellipodia and its activation by S1P are essential in regulating PDGF-induced chemotaxis in U251 glioblastoma cells (Lepannetier et al., 2016).

Another member of the TRPC subfamily specifically implicated in glioma progression is TRPC6. Indeed, TRPC6 was found to affect different hallmarks of GBM including tumor growth, cell survival, invasiveness and angiogenesis (Chigurupati et al., 2010; Ding et al., 2010). More specifically, it has been demonstrated that under hypoxia, which is one of the main characteristics of GBM aggressiveness, invasion and resistance to treatment (Flynn et al., 2008), Notch1 activation consequently led to TRPC6 upregulation in primary GBM samples and cell lines (Chigurupati et al., 2010). Indeed, the inhibition/silencing of TRPC6 was associated with a reduction in glioma growth, invasion and angiogenesis. This Notch1-mediated induction of TRPC6 expression in hypoxic U373 cell line was subtype-specific, since other members of the TRPC subfamily were unaffected, indicating that TRPC6 is primarily responsible for the hypoxia-induced sustained increase in the intracellular Ca^2+^ concentration. Additionally, it has been proven that TRPC6 is essential for GBM cell survival, since its downregulation not only suppressed cell growth *in vitro* and reduced tumor volume *in vivo*, but also impaired clonogenic ability, induced cell cycle arrest at the G_2_/M phase, and enhanced the antiproliferative effect of ionizing radiation.

An accelerated G_2_ phase progression may lead to an impaired DNA damage checkpoint and thus to an enhanced genomic instability, explaining the TRPC6 association with enhanced glioma cell malignancy. Mechanistically, TRPC6 effects on cell proliferation, tumor growth and angiogenesis seemed to be directly mediated by the Ca^2+^-induced activation of the calcineurin-NFAT pathway (Chigurupati et al., 2010), whereas TRPC6 effects on cell migration might rather involve Rho activation and subsequent actin cytoskeleton rearrangements (Singh et al., 2007). Together, these data stress a possible role of TRPC6 as a promising therapeutic target in the treatment of human GBM (Chigurupati et al., 2010; Ding et al., 2010).

### Melastatin TRPs

Four TRP melastatin subfamily members, TRPM2, TRPM3, TRPM7, and TRPM8, have been implicated in glioma cell growth, proliferation and migration. Among them, TRPM2 and TRPM3 would exert anti-tumorigenic effects, while TRPM7 and TRPM8 may contribute to glioma malignancy.

TRPM2 is known for its role as a sensor of oxidative stress and inductor of necrotic cell death upon activation by reactive oxygen species (ROS) (Naziroğlu and Lückhoff, 2008; Takahashi et al., 2011). TRPM2 expression likely induced no effect on cell proliferation, migration and invasion. But in A172 human GBM cells the transfection with TRPM2 channels increased cell death induced by H_2_O_2_ in a Ca^2+^-dependent manner (Ishii et al., 2007) and in human GBM (DBTRG) cells, TRPM2 activation led to an increase in oxidative stress and intracellular Ca^2+^ concentration, thus promoting GBM cell death through apoptosis (Ertilav et al., 2019). Concerning TRPM3, which is the most recently described melastatin subfamily member, Its roles and action mechanisms were only recently investigated (Zamudio-Bulcock et al., 2011). Like TRPM2, TRPM3 may show a protective role in glioma probably via the miR-204 regulation (Ying et al., 2013), but its function needs further studies.

TRPM7 also controls glioma progression through miRNA regulation in GBM cells with subsequent effects on cell proliferation and invasion (Wan et al., 2019). More specifically, TRPM7 expression can be associated with a decreased production of miR-28-5p, a tumor suppressor inhibiting the expression of oncogenic signaling pathways involving protein-kinase B (AKT) (Xiao et al., 2018), ERK (Liu et al., 2016), and IGF-1 (Shi and Teng, 2015). Accordingly, the downregulation of miR-28-5p caused a significant increase in glioma cell proliferation and invasion. Among miR-28-5p targets, expression of Rap1b appeared as being positively correlated with TRPM7 in GBM and was up-regulated in tumor samples due to the suppression of the repressing role of miR-28-5p (Wan et al., 2019). This miR-28-5p/Rap1b axis shown in gliomagenesis is not the exclusive signaling route in which TRPM7 acts on GBM progression. Indeed, TRPM7 may also regulate the Notch pathway (Liu et al., 2014), recently shown as linked with Rap1b signaling and integrin-mediated cell adhesion in hematopoietic stem cells (Rho et al., 2019). This can be connected with the role of TRPM7 on cell proliferation, migration and invasion in glioma cells and GSCs through the upregulation of JAK2/STAT3 and/or Notch signaling pathways (Liu et al., 2014). Moreover, TRPM7 was found to activate STAT3, which in turn binds to the aldehyde dehydrogenase1 (ALDH1) promoter upregulating the expression of this well-known GSC marker involved in many pathways maintaining stem cell-like state (Rasper et al., 2010), when expanded as spheroids (Liu et al., 2014). Since ALDH1 is functionally involved in self-protection, differentiation, expansion and proliferation (Choudhary et al., 2005; Ma and Allan, 2011), this potentially means that TRPM7 is not only implicated in proliferation, migration and invasion, but also in GSC renewal and differentiation. This has to be put into the context that TRPM7 channel exhibits an intrinsic kinase activity, thus supporting that TRPM7 effects on glioma cell growth are mediated by its channel activity while cell migration and invasion required its kinase domain (Wan et al., 2019). The discovery of different cellular and molecular targets affecting gliomas development and progression through their modulation by TRPM7 provides key insights for the development of novel therapeutic agents for glioma treatments.

TRPM8 was found to affect the rate of GBM cell migration by mediating a significant increase in intracellular Ca^2+^ concentration upon stimulation with specific agonists such as menthol and icilin (Wondergem et al., 2008; Wondergem and Bartley, 2009; Klumpp et al., 2017). It has been shown that TRPM8 activation by icilin leads to a significant increase in the migration speed and chemotaxis of GBM cells and, consistently, TRPM8 downregulation by RNA interference as well as TRPM8 inhibition by the specific channel blocker BCTC [*N*-(4-tertiarybutylphenyl)-4-(3-cholorphyridin-2-yl)tet rahydropyrazine-1(2H)-carbox-amide] reduces cell migration rate and decreases transfilter chemotaxis (Klumpp et al., 2017). One of the possible mechanisms through which TRPM8-mediated Ca^2+^ influx may affect cell migration in glioma is by the activation of the large-conductance Ca^2+^-activated K^+^ ion channels (BK channels) (Wondergem and Bartley, 2009). BK channels contribute to maintaining the plasma membrane ionic fluxes essential to support cell shrinkage-driven cell migration (Mcferrin and Sontheimer, 2006). Interestingly, BK overexpression was detected in human glioma cells (Ransom and Sontheimer, 2001) and pharmacological inhibition of BK channels was shown to abolish the menthol-stimulated Ca^2+^ influx within the cell cytoplasm and cell migration suggesting a key role of TRPM8. TRPM8 activation by agonists has been shown to increase the open probability of single BK channels (Wondergem and Bartley, 2009; Klumpp et al., 2017). In agreement, ionizing radiation, known to induce the migration through a Ca^2+^-mediated activation of BK channels (Steinle et al., 2011; Edalat et al., 2016), has been shown to activate and upregulate TRPM8-mediated Ca^2+^ influx in glioma cells (Klumpp et al., 2017), thus confirming a direct and reciprocal interplay between these two channel families in the control of GBM migration. The function of TRPM8 in aggressiveness and resistance to treatment was suggested by the potentiating impact of hepatocyte growth factor/scatter factor (HGF/SF), a multifunctional effector of cells expressing the Met tyrosine kinase receptor (TKR), on TRPM8-induced Ca^2+^ homeostasis and cell migration. This evidence suggests that TRPM8 might converge to a common HGF/SF and cMET, known to play a role in malignancy of solid tumors including glioma (Laterra et al., 1997; Birchmeier et al., 2003; Wondergem et al., 2008), signaling pathway leading to migration/invasion. An enhancement in the invasion rate of human GBM cells has also been associated with TRPM8 overexpression (Zeng et al., 2019). However, DBTRG cells would express two different variants of TRPM8 (Wondergem et al., 2008) as revealed in Western blot showing a molecular band at 130–140 kDa in the plasma membrane-enriched fraction and consistent with the molecular weight of TRPM8 full-length isoform (Peier et al., 2002), and a second molecular band at 95–100 kDa in microsome- and membrane-enriched fractions more consistent with a truncated TRPM8 splice variant expressed in the endoplasmic reticulum (ER) (Bidaux et al., 2007). The observed greater increase in menthol-induced Ca^2+^ influx among migrating cells compared with non-migrating cells (Wondergem et al., 2008), likely indicates that only migrating cells express full-length TRPM8 protein within the plasma membrane. However, these results have to be taken with caution in a future context of drug therapy, since TRPM8 was shown to have an anti-migratory activity in other cancers including prostate cancer and may play a role in the tumoral and tumor-derived endothelial cells (Gkika et al., 2010, 2015; Genova et al., 2017; Grolez et al., 2019b). TRPM8 contribution to GBM progression was found to go far beyond its effects on cell migration and invasion, significantly affecting other determinant processes such as cell cycle, cell survival and radioresistance (Klumpp et al., 2017; Zeng et al., 2019). Indeed, it has been proven that TRPM8 inhibition or knockdown impaired the cell cycle, triggered apoptotic cell death and attenuated DNA repair and clonogenic survival (Klumpp et al., 2017). A recent study by Zeng et al. (2019) have suggested an involvement of the mitogen-activated protein kinase (MAPK) signaling pathway in TRPM8-mediated effects on cell proliferation and apoptosis, since the expression of the channel was associated with the expression levels of important regulators of these pathways, including extracellular signal-regulated kinase (ERK), cyclin D1 and the apoptosis-related protein Bcl-2 in human glioma cells. Moreover, it has been shown that TRPM8 signaling directly regulates the cell cycle, contributing to S phase progression and mitosis. These effects on glioma’s cell cycle are most likely mediated by intracellular signaling pathways involving the Ca^2+^/calmodulin-dependent protein kinase II (CaMKII) and some Cdc phosphatases like Cdc25C and Cdc2, which control entry into, and progression through, various phases of the cell cycle (Klumpp et al., 2017). More specifically, the TRPM8-mediated Ca^2+^ entry, through the activation of BK channels, may increase the CaMKII activity, which in turn inhibits the Cdc2 subunit of the mitosis-promoting factor likely through the inhibitory phosphorylation of the Cdc25C phosphatase (Klumpp et al., 2017). Finally, TRPM8 channel might also modulate proliferation by dynamically control glioma resting potentials levels, which are key regulator of cell cycle. TRPM8 agonists have been shown to increase the Kir4.1 mediated membrane conductances of glioma cells (Ratto et al., 2020). Kir4.1 is an inward rectifier K^+^ channel, with an altered pattern of expression in glioma. In astrocytes, it is responsible for high potassium conductance and hyperpolarized membrane potential (Olsen and Sontheimer, 2008). Overexpression of this channel in glioma cell lines D54MG reduces cell proliferation (Higashimori and Sontheimer, 2007). Thus, TRPM8 channel by regulating K^+^ resting conductances of glioma cells may exert a regulation of cell cycle transitions. Moreover, TRPM8 has also been found to be a contributor to the genotoxic stress response of GBM upon treatment with ionizing radiation, restoring G_1_/S transition and S phase progression to levels of unirradiated cells (Klumpp et al., 2017). Interestingly, it has been found that ionizing radiation stimulated TRPM8 availability both *in vitro* and *in vivo* and that TRPM8 played a role in the re-entry in mitosis and cell division upon radiation-induced G_2_/M arrest, since its knockdown resulted in an impaired DNA repair and a decreased survival of irradiated cells (Klumpp et al., 2017). This, combined with the slowdown of apoptosis in irradiated GBM cells, may explain the enhanced radioresistance acquired by GBM cells overexpressing TRPM8, thus stressing that the key interest of targeting TRPM8 alone or in combination with radiotherapy for future treatments of GBM.

### Vanilloid TRPs

Some TRP members of the vanilloid family have been related to gliomagenesis and progression. More specifically, TRPV1 and TRPV2 have revealed a protective role in glioma cells by regulating cell proliferation and survival, stem cell differentiation and sensitivity to drugs, whereas TRPV4 was found to increase cancer cell invasiveness.

The anti-tumorigenic functional role of TRPV1 in gliomas, suggested by its marked downregulation or loss in patients with the shortest overall survival, has been investigated and several findings highlighted a role of TRPV1 in the induction of apoptotic cell death signaling in gliomas (Amantini et al., 2007; Stock et al., 2012). Upon exposure to low doses of the TRPV1 specific agonist capsaicin (CPS), the TRPV1-Ca^2+^ may sustain apoptosis in gliomas through the selective activation of p38 MAPK, but not ERK MAPK (Amantini et al., 2007). More in detail, it has been shown that CPS-mediated TRPV1 activation leads to reduced cell viability, DNA fragmentation, externalization of phosphatidylserine on the outer layer of the plasma membrane, mitochondrial transmembrane potential dissipation, and caspase 3 activation (Amantini et al., 2007). Moreover, TRPV1 translation in GBM was found to be sensitive to interferon-gamma (INF-γ) and to the well-known autophagic inducer rapamycin (Rap), suggesting a link between TRPV1 channel and autophagy often related to pro-survival in tumors including GBM (Galluzzi et al., 2015) but also in migration (Coly et al., 2017). This biological effect might be achieved by the TRPV1-mediated induction of apoptosis previously reported in GBM cells (Amantini et al., 2007).

One of the mechanisms through which the brain, especially in the juvenile phase, can protect itself against high-grade astrocytoma (HGAs) involves the activation of TRPV1 by neural precursor cells (NPCs), known to show extensive tropism for brain tumors (Mayor et al., 2001). Interestingly, NPCs accumulate at HGA especially in the context of the juvenile brain, exhibiting a high proliferative activity in the stem cell niche (Walzlein et al., 2008), and can release tumor-suppressive factors, such as endovanilloids able to activate TRPV1 expressed by HGA cells (Stock et al., 2012). The activation of the latter would trigger astrocytoma cell death through the ER pathway in a transcription factor-3 (ATF3)-dependent manner, thus reducing glioma expansion mostly in young brain (Stock et al., 2012). In light of these data, the inverse correlation between TRPV1 expression and glioma grade from I to III and the reduced or lost TRPV1 expression found in GBM patients is most likely a mechanism by which tumor cells may evade anti-proliferative and pro-apoptotic signals. This hypothesis is also supported by the finding that TRPV1 is also downregulated in GSCs (Stock et al., 2012), whose resistance to cytotoxic therapies and to pro-apoptotic signals is accepted (Bao et al., 2006a). Furthermore, the induction of GSCs differentiation was accompanied by TRPV1_*v*__3_ expression at a similar level than found in low-grade glioma, thus confirming a protective role of this channel against aggressiveness (Nabissi et al., 2016).

TRPV2 exerts its anti-tumorigenic function on gliomas through the regulation of several signaling pathways involved in cell proliferation and survival, stem cell differentiation and sensitivity to drugs. Physiologically, the triggering of TRPV2 by agonists/activators such as growth factors, hormones and cannabinoids led to TRPV2 translocation from the endosome to the plasma membrane, where it mediates several pathways associated with cell proliferation and cell death (Liberati et al., 2014). Thus, loss or alterations of TRPV2 expression in cancer cells results in an impairment of these processes, as shown in prostate tumor-derived endothelial cells (Bernardini et al., 2019) and gliomas (Liberati et al., 2014). In gliomas, it has been shown that TRPV2 reduced cell proliferation and increased cell sensitivity to Fas-induced apoptosis in an ERK-dependent manner (Nabissi et al., 2010). Consistently, enhanced cell growth and rescuing from apoptotic cell death was observed when TRPV2 was silencing in U87MG GBM cells. In contrast, TRPV2 upregulation in MZC primary glioma cells, by inducing Fas overexpression led to reduced viability and increased spontaneous as well as Fas-induced apoptosis (Nabissi et al., 2010). Similar findings were also described in GSCs, whose proliferation appeared strongly impaired by TRPV2 pharmacological inhibition or knocking down (Morelli et al., 2012). In GSCs, TRPV2 acts also on differentiation (Morelli et al., 2012; Nabissi et al., 2015). More specifically, TRPV2 overexpression was associated with glial fibrillary acidic protein (GFAP) and β_III_-tubulin increased expression, thus promoting a glial phenotype differentiation while inhibiting GSCs proliferation both *in vitro* and *in vivo.* In agreement, TRPV2 silencing or inhibition during differentiation impaired differentiation and reduced GFAP and β_III_-tubulin expression (Morelli et al., 2012). Moreover, TRPV2 activation through cannabidiol (CBD) was found to trigger GSCs differentiation activating the autophagic process, in addition to inhibiting GSCs proliferation and clonogenic capability (Nabissi et al., 2015). More specifically, it has been observed that CBD, through the TRPV2-mediated activation of the PI3K/AKT pathway, upregulated the expression of acute myeloid leukemia (Aml-1) transcription factors, known for their pivotal role in GBM proliferation and differentiation. Furthermore, it has also been shown that the spliced variant Aml-1 a, upregulated during GSCs differentiation, directly influenced the expression of TRPV2 by binding its gene promoter (Nabissi et al., 2015), thus establishing a positive feedback circuit, which on the whole caused glial differentiation.

Conversely to TRPV1 and TRPV2, TRPV4 has revealed a pivotal role in promoting glioma progression (Ou-yang et al., 2018). In particular, the tumorigenic potential of TRPV4 comes from its critical involvement in glioma cell migration and invasion. Indeed, it has been demonstrated that TRPV4-mediated Ca^2+^ influx upon stimulation with the selective agonist GSK1016790 A, is able to promote cell migration of glioma cells (Ou-yang et al., 2018), a similar mechanism previously reported in breast cancer (Lee et al., 2017). It was established that TRPV4 effects on cell migration are relayed by phosphorylation of AKT (P-AKT) and activation of Rac1 (Ou-yang et al., 2018), a member of the Rho GTPases family known for its central role in cytoskeleton remodeling, cell motility and cell adhesion as well as for its involvement in the enhanced migration of several tumor types including GBM, colon, colorectal and ovarian cancer (Guo et al., 2015; Guéguinou et al., 2016; Qin et al., 2017; Zhou et al., 2018). Accordingly, TRPV4 blockade, induced by the specific TRPV4 inhibitor HC-067047, was found to decrease motility and invasiveness of U87 glioma cells through a P-AKT and Rac1 signaling pathway (Ou-yang et al., 2018).

### Mucolipin TRPs

The two TRP members of mucolipin subfamily have revealed opposite effects on glioma carcinogenesis.

TRPML1 showed a protective role against glioma progression. Indeed, it has been shown that TRPML1 activation by its specific agonist MK6-83 reduced T98 and U251 cell line viability and increased caspase 3-dependent apoptosis. Accordingly, TRPML1 silencing or pharmacological inhibition restored cell viability suppressing the Ca^2+^ influx responsible for apoptosis induction. Furthermore, TRPML1 may also mediate the autophagic cell death pathway, upon cell treatment with the ROS inducer carbonyl cyanide *m*-chlorophenylhydrazone (CCCP) (Morelli et al., 2019). Indeed, high ROS levels may trigger a TRPML1-mediated lysosomal Ca^2+^ release and the subsequent enhancement of autophagy (Zhang et al., 2016b). Accordingly, TRPML1 silencing or inhibition by sphingomyelin pre-treatment reverted CCCP effects (Morelli et al., 2019). In this context, it has also been demonstrated that TRPML1, through a large intraluminal loop between its first and second transmembrane domains, may interact with chaperone-mediated autophagy-related proteins such as the heat shock proteins Hsc70, and Hsp40 (Venugopal et al., 2009). Therefore, TRPML1 may exert its cytotoxic effects through two different pathways, e.g., (i) it can act as a ROS sensor on the lysosomal membrane and attenuate oxidative cell stress through an autophagy-dependent negative-feedback mechanism (Zhang et al., 2016a; Morelli et al., 2019) or (ii) it may trigger Ca^2+^ release but no ROS production upon direct activation by its specific agonist, thus inducing apoptosis (Morelli et al., 2019). Moreover, the important role of TRPML1 in controlling intracellular Ca^2+^ homeostasis has been further corroborated by the recent finding of a functional localization of TRPML1 at the so-called “mitochondria-lysosome contact sites” where it mediates a calcium flux from lysosomes to mitochondria adjuvanted by VDAC and MCU on the outer and inner mitochondrial membranes, respectively (Peng et al., 2020). Consequently, TRPML1 functions go beyond the regulation of lysosomal dynamics and function and, through the control of mitochondrial Ca^2+^ dynamics, can affect other Ca^2+^-dependent mitochondrial functions, including oxidative phosphorylation, motility, and ROS signaling (Peng et al., 2020).

Conversely to TRPML1, the other member of the mucolipin family, TRPML2, has revealed a pro-tumorigenic function in glioma progression. Indeed, it has been shown that TRPML2 enhanced glioma cell survival and proliferation (Morelli et al., 2016). More in detail, TRPML2 suppression leads to impaired cell cycle, reduced cell viability and decreased proliferation (Morelli et al., 2016). In addition, its knocking-down was found to induce apoptosis by increasing DNA damage, Ser139 H2AX phosphorylation and caspase-3 activation (Morelli et al., 2016). TRPML2 effects on tumor progression are probably mediated by PI3K/AKT and ERK1/2 pathways, since these pathways remained inactivated in TRPML2-silenced cells (Morelli et al., 2016). Thus, TRPML2 might also be an interesting therapeutic target to control GBM cell survival and proliferation.

## Therapeutic Targeting

Considering the altered expression and the great contribution given by TRP channels to the establishment and progression of glioma, they may be considered very promising new therapeutic molecular targets against which novel drug compounds must be developed. One of the main advantages provided by most TRPs is their accessibility from the extracellular side, which makes them efficient targetable sites via administration of specific TRPs inhibitors or blockers when these channels are overexpressed in high-grade glioma. For instance, some of the TRPC channels by interfering with cytokinesis pathways would be promising targets for the development of drugs able to interfere with the almost unrestrained growth of gliomas, making tumors more susceptible to surgical removal or focal radiotherapy. Moreover, the low specificity of some TRPC modulators might allow to achieve a higher antitumor effect, through the simultaneous triggering of more than one channel. The progressive understanding of the molecular mechanism underlying TRPC function in glioma has also provided opportunities and arguments in favor of small molecule targeted therapies. Through studies concerning TRPC1 and its specific pharmacological inhibition, the key option by inhibiting SPK or PI3K inhibitors is once again here confirmed to attempt controlling GBM growth and invasiveness. Among TRPC channels, great potential as a promising new candidate for GBM treatment comes from TRPC6. Among TRP, TRPC6 is to date the only one being implicated in GBM angiogenesis suggesting that specific TRPC6 inhibitors could simultaneously target both cancer progression and vascularization, thereby improving the efficacy of standard (radio-chemotherapy) options. The barely detectable TRPC6 expression in normal glial cells should limit the potential side effects on normal glial cells. But a possible impact of TRPC6 blockade on neurons must be considered in regards to the central role of TRPC6 in neuronal functions (Tai et al., 2008; Zhou et al., 2008; Kim et al., 2017). A possible strategy to overcome this problem might be the use of viral vectors as a drug delivery system toward glioma cells since adenovirus may target glioma cells more efficiently than neurons (Ding et al., 2010).

As recurrence is due to migration and invasion (and resistance to treatment), pharmacological inhibition of TRPV4 might represent a potential new therapeutic approach in GBM treatment to control migratory and invasive capabilities of GBM cells (Ou-yang et al., 2018). One of the mechanisms through which tumor cells are able to sustain a prolonged survival is by the inhibition of apoptotic pathways (Thompson, 1995). In this context, TRPA1 has been recently proposed as a new potential therapeutic target in GBM treatment. Indeed, TRPA1 is a ROS-sensitive cation channel and can subsequently be activated by hypoxia-induced oxidative stress (Naziroğlu, 2012; Takahashi et al., 2018). It has been shown that TRPA1 activation following cobalt chloride (CoCl_2_) treatment with the aim to mimic hypoxia, may increase apoptosis, inflammation and oxidative effects on DBTRG cells (Deveci et al., 2019). More specifically, TRPA1-mediated Ca^2+^ entry was associated with an enhancement of ROS production and mitochondrial membrane depolarization (JC-1). Moreover, TRPA1 activation leads to increased levels of Annexin V, cytokines IL-1β and IL-18, and caspase 3 and 9 and decreased levels of thiol cycle antioxidants (GSH and GSH-Px) (Deveci et al., 2019). These effects were shown attenuated by α-lipoic acid (ALA) treatment, a physiological source of energy for cells which may exert both anti- and pro-oxidant functions (Moini et al., 2002). In glioma cells under hypoxia, ALA likely acts as an antioxidant agent, upregulating GSH and GSH-Px and down-regulating mitochondrial ROS production, thus blocking TRPA1-mediated induction of apoptotic cell death (Deveci et al., 2019). This suggests that targeting and activating TRPA1 or targeting TRPV1 in glioma exhibiting such expression, can restore apoptotic signaling and might provide new insights for the development of alternative therapies against glioma progression.

Besides being potential anti-tumor targets, TRP channels should play a role as “drug carriers” in cancer therapy, facilitating *via* the central pore chemotherapy drug uptake thus improving the efficacy of cancer therapies. For instance, TRPV2 activation by CBD can sensitize GBM cells to chemotherapeutic agents currently used, e.g., TMZ, carmustine (BCNU) and doxorubicin (DOXO) (Nabissi et al., 2013, 2015). The CBD-induced TRPV2 activation was found to increase GSCs sensitivity to cytotoxic effects of alkylating agents like BCNU favoring drug uptake (Nabissi et al., 2013), in synergy with the Ca^2+^-dependent triggering of apoptotic cell death, a mechanism not found in normal astrocytes (Nabissi et al., 2015). Specifically, by using the natural red fluorescent DOXO, it has been demonstrated that TRPV2 overexpression in MZC glioma cells markedly increased DOXO uptake in a Ca^2+^-dependent manner, since Ca^2+^ chelation by EGTA completely inhibited the CBD-induced TRPV2-mediated increase of DOXO-positive cells (Nabissi et al., 2013). Similar findings were also observed in hepatocellular carcinoma in which TRPV2 activation by CBD or 2-APB (Aminoethoxydiphenyl borate) was found to improve DOXO permeation into tumor cells, thus corroborating an intriguing role of TRPV2 in increasing tumor cell sensitivity to chemotherapy drugs (Neumann-Raizel et al., 2019). Taking into account other evidence on the role of TRP channels as “drug carriers” thanks to the permeation of chemotherapy agents into the cell through their pore domain (Santoni and Farfariello, 2011), it is reasonable to speculate that the activation of TRPV2 channel may cause a conformational change in the pore helix structure, which allow for intracellular non-specific chemotherapy uptake (Nabissi et al., 2013), opening the route for combinatorial co-administration of TRPV2 specific agonist CBD and lower chemotherapeutic doses to overcome the high resistance of GBM and GSCs to chemotherapeutic agents. In this context, recent developments on the role of the so-called “pore turret,” i.e., the region of the extracellular ring that connects the S5 helix to the pore helix, in controlling the upper gate of some TRP channels including TRPV2 (Dosey et al., 2019) have provided new insights into the role of TRPV2 as a drug target to reduce GBM chemoresistance. Indeed, the well-defined pore turret, in addition to allowing the coupling between the lower gate and the upper gate in response to intracellular stimuli stabilizing a fully open unliganded channel, represents a possible and interesting binding site for extracellular modulators through which it may affect channel activity allowing the passage through the plasma membrane of partially hydrophilic molecules which otherwise could not enter the cell (Dosey et al., 2019).

With a view to looking ahead, some data indicate that TRP channels may constitute targets of gene therapy. It has been recently shown that TRPM2 can promote cell death (Ertilav et al., 2019). Therefore, TRPM2 might represent a good candidate for gene therapy to be used for instance in combination with γ-radiation and/or chemotherapeutic agents to improve the effectiveness of GBM treatments. Preliminary observations indicate that Selenium (Se) tested on GBM cells resistant to Docetaxel (DTX) may improve the apoptotic efficacy of DTX through the activation of TRPM2 by oxidative stress (Ertilav et al., 2019). The cytotoxic effect of DTX likely comes from the formation of excessive mitochondrial ROS and Ca^2+^ influx into the cells which causes DNA damage by triggering hyperactivation of the DNA nick sensor PARP, thus leading to NAD+ and ATP depletion and subsequent apoptotic cell death (Ertilav et al., 2019). In this case, Se, in particular, stimulated oxidative stress production in the mitochondria, which in turn activated a TRPM2-mediated Ca^2+^ influx, thus supporting and enhancing the same Ca^2+^-dependent apoptotic pathway induced by DTX and other chemotherapeutic agents, as seen also in other tumor types (Hazane-Puch et al., 2016; Çetin et al., 2017). Overall the combination and synergistic activity of Se and DTX in GBM expressing TRPM2 might offer a new option for adjuvant chemotherapy as treatment of GBM.

## Future Perspectives

Increasing understanding of the signaling pathways involved in tumorigenesis has made it possible to identify a wide range of molecular targets involved in self-renewal and proliferation, angiogenesis but also in invasion of GBM cells. A number of therapeutic strategies have therefore been developed during the last decade and few of them have proven to be effective, even though anti-angiogenic treatments appear to be able to provide a 6 months delay for GBM patients before recurrence. It is, therefore, necessary to identify other therapeutic targets that can be combined with anti-angiogenic, cytotoxic, DNA repair inhibitors and/or immunotherapy strategies. In this context, targeting the activity of factors or components expressed by glioma cells themselves and by other cell types of the micro-environment would also be promising. It has also to be considered that main RNAseq and/or transcriptomic databases were constituted by means of the glioma tumor bulk composed of the different populations of GBM cells and other constituents such as endothelial cells, pericytes, reactive astrocytes, macrophages (M1 and/or M2), microglial, neurons, and potentially lymphocytes, depending on the level of heterogeneity GBM subgroup. New potential family targets, expressed at the plasma membrane and involved in survival, GSC differentiation, angiogenesis and invasion, constitute a choice option. TRP channels not systematically ubiquitously expressed, potentially playing pleiotropic mechanisms and being overexpressed in pathologic situations such as hypoxia in glioma cells deserve to be more explored, especially since they could be also expressed by other cell types belonging to the tumor micro-environment.

For instance, a direct involvement of TRP channels in vascular endothelial growth factor (VEGF) signaling pathways affecting brain neovascularization and tumor growth it has been proven. GCSs positive for CD133 (human prominin-1/AC 133) not only are capable of self-renewal and proliferation, but also possess the capability to secrete high levels of VEGF (Bao et al., 2006b; Yao et al., 2008), known to play a crucial role in endothelial cell recruitment and angiogenesis of malignant human gliomas (Fischer et al., 2005; Bian et al., 2006). In GSCs isolated from U87 cell line the production of VEGF and the angiogenic CXCL8 (chemokine interleukin-8) by tumor cells appeared to be mediated by a G protein-coupled receptor named formylpeptide receptor (FPR) which, upon activation, induces directional migration, growth and angiogenic factors production through a Ca^2+^ mobilization. Although a direct involvement of TRPs in this Ca^2+^-mediated mechanism has not been highlighted, a GCPR-TRP axis in many signaling pathways is nowadays well established (Veldhuis et al., 2015). Moreover, an interesting crosstalk between TRPM8, TRPV1 and the VEFG receptor (VEGFR) it has been recently characterized in uveal melanoma, suggesting a good potential for TRPM8 as a pharmacological target for blocking brain neovascularization and tumor growth (Walcher et al., 2018). Indeed, it has been demonstrated that in different cell types including corneal epithelial and endothelial cells (Lucius et al., 2016) and uveal melanoma cells (Walcher et al., 2018) the activation of TRPM8 inhibits the VEGF transactivation of TRPV1 and the consequent pro-tumorigenic effects mediated by the VEGFR. These findings further highlighted the central role played by TRPs interactions with other TRP channels, other channels families like that of BK channels, and the GCPRs, in affecting signaling pathways directly involved in carcinogenesis and brain tumor progression. Moreover, they strongly sustain the possible TRPs application in anti-angiogenic therapy.

However, to date, to the best of our knowledge TRPC6 is the only TRP channel directly implicated in GBM angiogenesis. TRPC6 has been found to play a key role in promoting GBM growth, angiogenesis and invasion under hypoxia through Notch1 (Chigurupati et al., 2010). TRPC6 knockdown or NFAT inhibition has been shown to reduce the number of branch points and thus impair the ability of the hypoxic U373MG to induce endothelial cell tube formation *in vitro*, suggesting a role of TRPC6 in the “vascular mimicry” played by glioma cells (Chigurupati et al., 2010).

Other TRPs among those previously described to have a function in gliomas, such as TRPM2, TRPM7, TRPV2, and TRPV4 have also been found in brain vasculature, thus suggesting a possible double function for these channels in affecting glioma progression (Hatano et al., 2013; Pires and Earley, 2017; Ou-yang et al., 2018; Luo et al., 2019) and contributing to GBM angiogenesis. Regarding TRPA1 and TRPC3 not likely described in glioma, some studies highlighted their involvement in other brain vasculature diseases, exerting a protective role against ischemic damage, controlling vasodilation in brain endothelial cells (Sullivan et al., 2016; Pires and Earley, 2018). TRPC3, when overexpressed, would lead to an increase in the BBB permeability, leading to vasogenic edema formation (Ryu et al., 2013).

## Conclusion

Taken together all these data suggest a key role of some TRP channels in high-grade glioma development and angiogenesis. The current activators or inhibitors directed against these channels should provide lead compounds and knowledge for future research in the design of drugs targeting simultaneously glioma cells and key components of the micro-environment such as abnormal tumoral vascularization.

## Author Contributions

DG and HC provided a rational of the study. GC performed an in-depth analysis of the roles of TRP channels in inducing glioma behavior and generated a preliminary draft. GC, HC, OC, and DG performed the literature searches and contributed to writing and editing of the content. All the authors contributed to the article and approved the submitted version.

## Conflict of Interest

The authors declare that the research was conducted in the absence of any commercial or financial relationships that could be construed as a potential conflict of interest.
